# Effect of individualized versus conventional perioperative blood pressure management on postoperative major complications in high-risk patients undergoing noncardiac surgery: study protocol for the SPROUT-4 multicenter randomized controlled trial

**DOI:** 10.1186/s13063-024-08707-4

**Published:** 2024-12-26

**Authors:** Jaeyeon Chung, Chang-Hoon Koo, Jungchan Park, Hye-Bin Kim, Jinyoung Bae, Jae-Woo Ju, Soowon Lee, Ah Ran Oh, Hyo Sung Kim, Soo Jung Park, Yunseok Jeon, Karam Nam, for the Seoul PeRioperative OUTcome research-4 (SPROUT-4) Investigators

**Affiliations:** 1 Department of Anesthesiology and Pain Medicine, Seoul National University Hospital, Seoul National University College of Medicine, 101 Daehak-Ro, Jongno-Gu, Seoul, 03080 Republic of Korea; 2 Department of Anesthesiology and Pain Medicine, Seoul National University Bundang Hospital, Seongnam, Gyeonggi Province Republic of Korea; 3 Department of Anesthesiology and Pain Medicine, Samsung Medical Center, Sungkyunkwan University School of Medicine, Seoul, Republic of Korea; 4 Department of Anesthesiology and Pain Medicine, Korea University Guro Hospital, Korea University College of Medicine, Seoul, Republic of Korea; 5 Department of Anesthesiology and Pain Medicine, Ajou University Medical Center, Ajou University of College of Medicine, Suwon, Gyeonggi Province Republic of Korea

**Keywords:** Anesthesia, Blood pressure, Hypotension, Postoperative complications, Randomized controlled trial

## Abstract

**Background:**

Intraoperative hypotension is very common during surgery and is linked to major organ dysfunction and mortality. Current perioperative blood pressure management is largely based on universal blood pressure thresholds ranging from a mean arterial pressure of 60–70 mmHg. However, the effectiveness of this conventional management remains unproven in prospective randomized trials. Therefore, we will conduct this study to test if individualized perioperative blood pressure management decreases the incidence of postoperative major adverse outcomes.

**Methods:**

This multicenter, randomized controlled superiority trial will enroll 1896 high-risk patients undergoing major noncardiac surgery from five tertiary university hospitals in South Korea. In the control group, mean arterial pressure will be maintained at ≥ 65 mmHg and systolic blood pressure ≥ 90 mmHg during surgery. In the intervention group, mean arterial pressure and systolic blood pressure will be maintained at no less than 20% of their baseline values. The baseline values are calculated as the average of all values measured from the day before surgery until the morning of surgery. These targets will be maintained until the patient is discharged from the post-anesthesia care unit or, for those who are transferred to the intensive care unit after surgery, until the end of the surgery. No specific restrictions, except for these blood pressure targets, will be applied to perioperative management. The primary composite outcome consists of all-cause death, stroke, myocardial infarction, new or worsening congestive heart failure, unplanned coronary revascularization, and acute kidney injury within 7 days after noncardiac surgery or until hospital discharge, whichever occurs first.

**Discussion:**

This study will reveal if individualized perioperative blood pressure management decreases the risk of major adverse outcomes in patients at high-risk undergoing noncardiac surgery.

**Trial registration:**

ClinicalTrials.gov NCT06225453. Registered on January 26, 2024.

**Supplementary Information:**

The online version contains supplementary material available at 10.1186/s13063-024-08707-4.

## Background

Intraoperative hypotension frequently occurs in noncardiac surgeries, with occurrences reaching nearly 70% [1]. Intraoperative hypotension is associated with various postoperative complications including acute kidney injury (AKI), myocardial injury, and death [2–4]. Despite the evident importance of perioperative blood pressure management, an optimal blood pressure management strategy has yet to be established. Instead, it is generally recommended to maintain the mean arterial pressure (MAP) above 60–70 mmHg, below which the risk of postoperative complications significantly increases [5]. Nevertheless, this widespread approach of aiming for a specific MAP threshold largely stems from retrospective observational studies [1, 3, 4, 6] and has not been proven effective in reducing postoperative complications in prospective randomized trials [7, 8].

Current guidelines advocate tailoring perioperative care based on patients’ associated conditions and comorbidities, particularly for those at high risk of postoperative complications [9, 10]. In this context, only one randomized controlled trial, the Intraoperative Norepinephrine to Control Arterial Pressure (INPRESS) study, showed that individualized perioperative blood pressure management could reduce postoperative organ dysfunction compared with the conventional strategy [11]. Yet, the methodology of the INPRESS study, which focused solely on systolic blood pressure (SBP) without considering MAP and aiming for perioperative SBP within 10% of the baseline level, diverges from the commonly accepted clinical practice of considering a 20% variance from the baseline [12]. Furthermore, the relatively small sample size (*n* = 298) hinders the broader applicability and interpretability of the findings in clinical settings.

We developed a multicenter randomized controlled trial with a refined methodology and a larger sample size to address these gaps. This study aims to test whether individualized blood pressure management effectively reduces postoperative complications compared with conventional management strategies among high-risk patients undergoing major noncardiac surgery.

## Methods

### Trial design

**Fig. 1:**
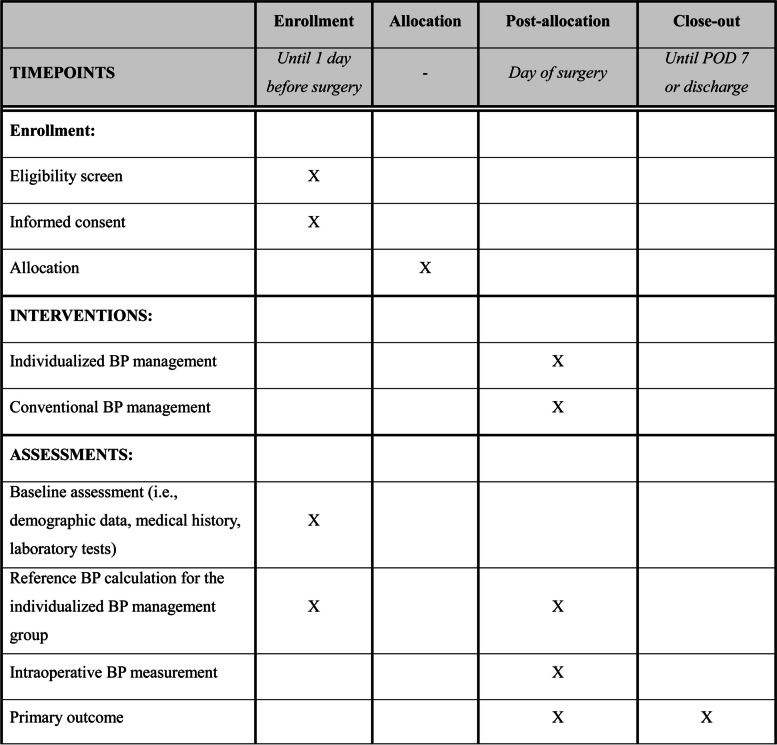
Study schedule for enrollment, interventions and assessments. POD, postoperative day; BP, blood pressure

The proposed Seoul PeRioperative OUTcome research-4 (SPROUT-4) trial will be a multicenter, parallel-group, randomized controlled superiority trial of patients undergoing elective major noncardiac surgery. We plan to enroll patients from five tertiary university hospitals in South Korea. The study protocol was approved by the Institutional Review Boards of the five participating hospitals (Ajou University Medical Center, No. AJOUIRB-IV-2024–042 on January 22, 2024; Korea University Guro Hospital, No. 2024GR0016 on January 4, 2024; Samsung Medical Center, No. 2023–12-098–001 on February 15, 2024; Seoul National University Bundang Hospital, No. B-2402–883-402 on February 2, 2024; Seoul National University Hospital, No. 2312–127-1496 on January 10, 2024) and registered at ClinicalTrials.gov on January 26, 2024 (NCT06225453). The trial coordinating center will be Seoul National University Hospital. The proposed trial will be conducted in compliance with the guidelines for Good Clinical Practice and the Declaration of Helsinki [13]. This manuscript adheres to the Standard Protocol Items: Recommendations for Interventional Trials statement (Fig. 1 and Additional file 1) [14].

### Patients

We will include high-risk patients scheduled for elective major noncardiac surgery under general anesthesia with an expected duration of more than 2 h. The high-risk criteria were determined based on a literature review [15]. Patients at a high risk are defined as those aged ≥ 65 years or those aged ≥ 45 years with more than one of the following: coronary artery disease, peripheral vascular disease, transient ischemic attack or stroke, or congestive heart failure. We will exclude patients undergoing emergency surgery, organ transplantation surgery, or brain or carotid artery surgery; patients with an American Society of Anesthesiologists physical status of 5 or 6; pregnant women; patients with uncontrolled hypertension, defined as SBP ≥ 180 mmHg or diastolic blood pressure ≥ 110 mmHg; patients with an estimated glomerular filtration rate < 30 ml/min/1.73 m^2^ or undergoing renal replacement therapy; patients with acute decompensated heart failure; or patients with sepsis, shock, or ongoing inotrope or vasopressor infusion.

### Informed consent

The Executive Committee, consisting of one investigator from each participating center, will be responsible for patient enrollment. After verification of the inclusion and exclusion criteria, written informed consent will be obtained from all patients. Patients will be informed about the study’s purpose and procedures and the expected harm and benefits of participation. The English version of model consent form is provided in Additional file 2. Patients will be notified that their participation is voluntary and that they are free to withdraw at any time without any disadvantage.

### Randomization and blinding

Patients who provide consent will be randomly assigned in equal numbers to either the individualized (intervention group) or conventional blood pressure management group (control group). Randomization will be conducted (R software, version 4.3.0; R Foundation for Statistical Computing, Vienna, Austria) by an independent research assistant using a block size of two, four, six, or eight and stratified by the hospital. Access to the randomization sequence will be secure, requiring a password, and will be revealed on the day of surgery. Although the nature of the interventions means that attending anesthesiologists will inevitably know each patient’s group assignment, the patients, surgeons, ward physicians, and statisticians analyzing the study data will not know which group they belong to.

### Protocol

**Fig. 2:**
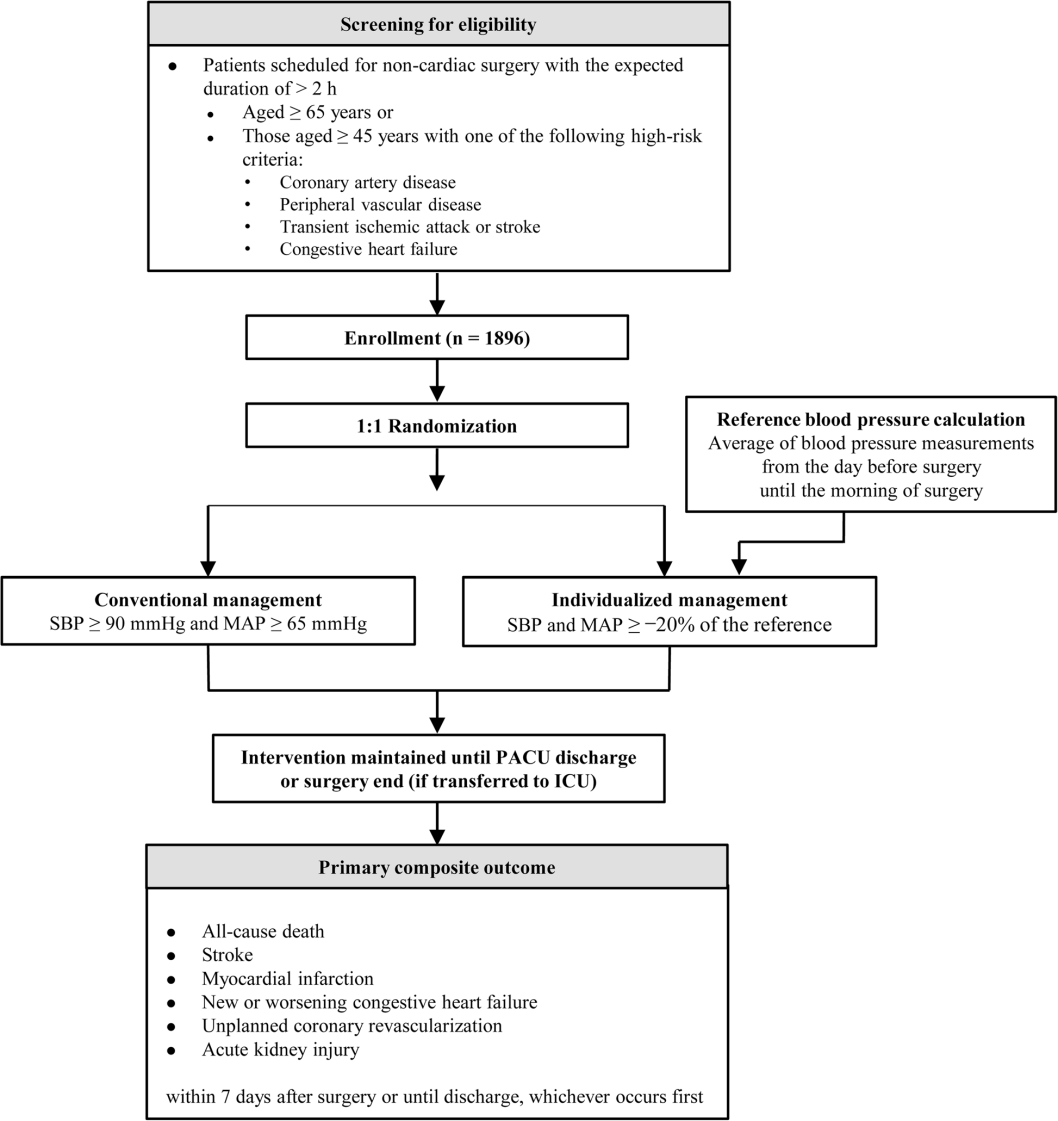
Flowchart of the SPROUT-4 multicenter, randomized controlled trial. SBP, systolic blood pressure; MAP, mean arterial pressure; PACU, post-anesthesia care unit; ICU, intensive care unit

A flowchart of the SPROUT-4 trial is shown in Fig. 2. The SPROUT-4 trial will not enforce specific restrictions on surgical procedures and perioperative management outside of the target blood pressure goals, thereby granting discretion to each participating hospital and attending anesthesiologist as well as closely mirroring real-world clinical practices. Consequently, there are no constraints on blood pressure measurement techniques (e.g., invasive arterial catheterization or noninvasive oscillometry), their application sites, measurement intervals, or the strategies implemented for maintaining designated blood pressure targets, including fluid and vasopressor administration, patient positioning, and anesthetic depth adjustment.

For the control group, the protocol dictates maintaining a MAP of at least 65 mmHg and an SBP of at least 90 mmHg throughout surgery for all patients. In the intervention group, MAP and SBP will be maintained at no less than 20% below baseline values. Baseline values will be calculated as the average of all MAP and SBP measurements obtained from the day before surgery until the morning of the surgery. These specified blood pressure targets are to be maintained until the patient is discharged from the post-anesthesia care unit or for those transferred to the intensive care unit after surgery until the end of surgery. For safety reasons, a minimum MAP of 50–55 mmHg will be maintained regardless of the calculated target values in the intervention group. This precaution ensures that blood pressure levels are kept within a safe range to mitigate the risk of hypotension-related adverse outcomes. If severe hypotension (defined as inability to maintain MAP above 50–55 mmHg despite interventions such as anesthetic adjustment, vasopressor administration, and fluid management) or any unexpected adverse events occur, the allocated intervention will be discontinued immediately, followed by appropriate therapeutic measures.

### Outcomes and definitions

The primary outcome will be a composite of all-cause death, stroke, myocardial infarction (MI), new or worsening congestive heart failure, unplanned coronary revascularization, and AKI. These outcomes will be assessed within 7 days after surgery or until discharge, whichever occurs first. The outcome components are defined as follows:

• Stroke: A new ischemic or hemorrhagic cerebrovascular accident with a focal neurological deficit confirmed using brain imaging
• MI: Diagnosed based on the Fourth Universal Definition of Myocardial Infarction (type 1, 2, or 3) [16]
  - ▪ Type 1 MI
    - ♦ Detection of a rise and/or fall of cardiac troponin levels with at least one value above the 99th percentile upper reference limit
    - ♦ In addition, one of the following is required:
      • Symptoms of acute myocardial ischemia
      • New ischemic electrocardiography (ECG) changes
      • Development of new pathological Q waves
      • Imaging evidence of a new loss of viable myocardium or regional wall motion abnormality in a pattern consistent with an ischemic etiology
      • Identification of a coronary thrombus by angiography, including intracoronary imaging or by autopsy
  - ▪ Type 2 MI
    - ♦ Detection of a rise and/or fall of cardiac troponin levels with at least one value above the 99th percentile upper reference limit
    - ♦ Evidence of an imbalance between myocardial oxygen supply and demand unrelated to coronary thrombosis
    - ♦ In addition, one of the following is required:
      • Symptoms of acute myocardial ischemia
      • New ischemic ECG changes
      • Development of new pathological Q waves
      • Imaging evidence of a new loss of viable myocardium or regional wall motion abnormality in a pattern consistent with an ischemic etiology
  - ▪ Type 3 MI
    - ♦ Cardiac death with symptoms suggestive of myocardial ischemia accompanied by presumed new ischemic ECG changes or ventricular fibrillation
    - ♦ In addition, one of the following is required:
      • Die before blood samples for biomarkers can be obtained
      • Die before increases in cardiac biomarkers can be identified
      • MI is detected by autopsy examination
• New or worsening congestive heart failure: Diagnosis on discharge letter of progression notes (medical records: pulmonary edema, congestive heart failure, etc.)
• Unplanned coronary revascularization: Percutaneous coronary intervention or bypass grafting, which was not an a priori planned stepwise procedure
• AKI is defined based on the serum creatinine criteria of the Kidney Disease: Improving Global Outcomes [17]: increase in serum creatinine level by 0.3 mg/dl or more within 48 h or to 1.5 times the baseline or more within 7 days. Baseline serum creatinine level is defined as the most recent preoperative value

The secondary outcomes will be individual components of the primary composite outcome, postoperative hospital length of stay, unplanned intensive care unit admission during index hospitalization, and new-onset atrial fibrillation of any duration, as captured by 12-lead ECG, continuous ECG monitoring, or telemetry, within 7 days after surgery or until discharge from the hospital, whichever occurs first.

### Sample size calculation and statistical analysis

Based on a pilot chart review conducted at the trial coordinating center (Seoul National University Hospital), the incidence of the primary outcome in the control group was estimated to be approximately 9%. We expected a 5% reduction in the incidence rate of the primary outcome in the intervention group. To detect this difference with an *α* of < 0.05 and a *β* of < 0.1 while assuming a dropout rate of 10%, we calculated that a total of 1896 participants, with 948 in each group, were necessary. There is no direct evidence in the literature regarding the expected 5% incidence of the primary outcome in the intervention group. However, we believe this is a conservative estimate. Should the benefits of this individualized approach prove significant, establishing such a conservative estimate ensures that any observed effects are robustly validated and attributable to the intervention itself, thereby minimizing the risk of overestimating its impact based on potentially confounding factors.

All analyses in the SPROUT-4 trial will be conducted in an intention-to-treat manner. Since the time window for all outcomes in this study is limited to the index hospitalization, complete follow-up is expected, with no anticipated missing data or loss to follow-up. Descriptive analyses will be performed to describe the baseline characteristics of the groups. The primary outcome will be compared between the groups using the chi-squared test. The relative risk and 95% confidence interval will also be reported. The same method will be applied to the binary secondary outcomes. The continuous secondary outcomes will be analyzed using Student’s *t*-test or Mann–Whitney *U* test, as appropriate. We will further compare the groups after excluding AKI from the primary composite outcome for sensitivity analysis. All analyses will be performed using the R software (version 4.3.0; R Foundation for Statistical Computing, Vienna, Austria). Statistical significance was set at *P* < 0.05.

### Data management and data safety monitoring

In the SPROUT-4 trial, data management and oversight will be conducted to uphold integrity and confidentiality. Patients’ data will be collected by dedicated trial assistant at each participating hospital. Unique patient identifiers will facilitate anonymized data analysis. All pseudonymized data will be retained in strict compliance with pertinent data protection regulations. These personnel, blinded to group assignments, will manage access to the data through a secure, password-protected file, ensuring impartiality and integrity in the handling of trial data. Each trial site will implement oversight mechanisms to protect participant rights and wellbeing; guarantee the precision, completeness, and verifiability of trial data; and ensure adherence to the trial protocol, Good Clinical Practice, and relevant regulatory standards.

A Data and Safety Monitoring Board, consisting of two cardiovascular anesthesiology and perioperative care specialists, will convene every 6 months to rigorously review the trial data. Their responsibilities include evaluating patient safety (occurrence of severe hypotension defined as MAP < 50–55 mmHg despite interventions or any unexpected adverse events), monitoring trial progress, and ensuring data integrity. They may suggest amendments to the study protocol or advise on its continuation or termination, based on the results. Any proposed protocol amendments will undergo a comprehensive review and will require approval from the Institutional Review Boards of all participating hospitals prior to implementation in the clinical trial. In the case of an expected or unexpected serious adverse event with a reasonable causal relationship to the trial protocol, participants will be compensated for both the resulting harm and any additional medical expenses incurred.

### Dissemination

The results of the SPROUT-4 trial will be reported to relevant scientific communities through publications in academic journals and presentations at national or international conference.

## Discussion

In this SPROUT-4 multicenter randomized controlled trial, we will evaluate whether individualized blood pressure management reduces the incidence of postoperative composite outcomes compared with conventional universal blood pressure threshold-based management. This outcome consists of all-cause death, stroke, MI, new or worsening congestive heart failure, unplanned coronary revascularization, and AKI that developed within seven days after major noncardiac surgery or until discharge, whichever occurs first.

The association between intraoperative hypotension and postoperative complications is well established [5, 18]. Indeed, large observational studies have demonstrated a strong relationship between intraoperative MAP of less than 55–65 mmHg and postoperative major cardiovascular morbidities, including AKI, myocardial injury, and death [1, 4, 19]. However, there remains considerable controversy over the optimal blood pressure levels in patients undergoing major noncardiac surgery to minimize major organ injury [20]. Surprisingly, a cause-effect relationship between blood pressure management and postoperative complications has not been proven in prospective randomized trials, further intensifying this controversy. Two recent randomized trials are worth noting: the Biomarkers, Blood Pressure, BIS: Risk Stratification/Management of Patients at Cardiac Risk in Major Noncardiac Surgery (BBB) study [8] and the PeriOperative Ischemic Evaluation-3 (POISE-3) study [7]. In the BBB study of 458 patients at cardiovascular risk, targeting intraoperative MAP ≥ 60 mmHg and ≥ 75 mmHg did not result in significant differences in postoperative outcomes [8]. The POISE-3 study of 7490 patients also failed to demonstrate any significantly different outcome between targeting intraoperative MAP ≥ 60 mmHg and ≥ 80 mmHg [7].

In contrast to previous studies, including the BBB and POISE-3 studies, which tested a couple of universal absolute MAP thresholds, the INPRESS study uniquely found that an individualized blood pressure management strategy, primarily utilizing the universal SBP threshold, significantly reduced the incidence of postoperative organ dysfunction compared with conventional management [11]. Unlike earlier studies, the INPRESS investigators maintained perioperative blood pressure within ± 10% of the reference value using norepinephrine in the individualized group. In contrast, the conventional group targeted SBP < 80 mmHg or a reduction of > 40% from the reference value using ephedrine first, followed by norepinephrine, if necessary. Nonetheless, several aspects of the INPRESS study underscored the need for the SPROUT-4 trial. First, the INPRESS study focused solely on SBP, with the reference value measured only once during preoperative anesthesiology consultation. Moreover, the strategies for maintaining the target blood pressure diverged between the groups (norepinephrine vs. ephedrine as the first-line vasopressor), which differs from real-world clinical practice that often employs a variety of methods, including different medications such as phenylephrine, vasopressin, patient positioning, and fluid bolus administration. Additionally, a relative difference of 10% was used for the individualized group in the INPRESS study, whereas a variance of 20% has been more commonly used [12]. Lastly, although the individualized group exhibited a significantly lower incidence of composite organ dysfunction, this difference was primarily driven by differences in AKI rates [11]. Given the low incidence of other major organ complications with a sample size of 298 patients, the statistical power of the INPRESS study may have been insufficient to discern differences in these other complications. To further address this issue, we will conduct the sensitivity analysis after excluding AKI from the primary composite outcome, similar to the BBB investigators [8]. Therefore, future studies including SPROUT-4 with refined methodologies are warranted.

To our knowledge, only one multicenter randomized trial, the IMPROVE-multi trial, is ongoing in 1272 high-risk patients undergoing major abdominal surgery in Germany to test individualized blood pressure management (ClinicalTrials.gov identifier, NCT05416944) [21]. The primary outcome will be a composite of AKI, MI, nonfatal cardiac arrest, and death within 7 days after surgery. Notably, in the IMPROVE-multi trial, the reference MAP for individualized management was based on the average nighttime blood pressure between 00:00 and 06:00 AM, measured at 30-min intervals. This approach to calculating the reference value is undoubtedly an improvement over the INPRESS study; however, applying this method outside strictly controlled research settings to real clinical situations seems impractical. Our SPROUT-4 trial method is more feasible for application in real-world clinical environments. Nevertheless, the results of these two studies are expected to overcome a significant portion of the limitations of the INPRESS study.

In this trial, the evaluation period for the primary outcome will focus on 7 days after surgery or until discharge. It is suggested that major organ injury typically occurs within 7 days of surgery [22, 23]. Furthermore, complications occurring beyond 7 days postoperatively are more likely to be attributed to other postoperative complications or the quality of post-surgical care, rather than blood pressure management during the perioperative period. While this approach provides clear insights into the effect of perioperative blood pressure management strategy on short-term outcomes, it does not capture long-term complications such as delayed mortality or major organ injury. Future research with a longer follow-up is warranted to provide a more complete understanding of the effects of the two different blood pressure strategies.

In summary, we will conduct a large multicenter randomized trial—with our pragmatic methodology and the largest sample size to date—to evaluate whether individualized perioperative blood pressure management reduces the incidence of a composite outcome of all-cause death, stroke, MI, new or worsening congestive heart failure, unplanned coronary revascularization, and AKI within seven days after surgery or until hospital discharge, whichever occurs first, compared to conventional blood pressure management in high-risk patients undergoing major noncardiac surgery.

## Trial status

The first patient was randomized on January 29, 2024. This manuscript was based on the first version of the protocol registered at ClinicalTrials.gov on January 26, 2024. Patient recruitment is estimated to be completed in September 2026.

## Supplementary Information

Additional file 1. SPIRIT Checklist.

Additional file 2. English version of model consent form.

## Data Availability

At the end of the trial, investigators will be allowed access to the data. Data will be available upon reasonable request, particularly for individual patient data meta-analyses.

## Abbreviations

AKI: Acute kidney injury
MAP: Mean arterial pressure
INPRESS: Intraoperative Norepinephrine to Control Arterial Pressure
SBP: Systolic blood pressure
SPROUT-4: Seoul PeRioperative OUTcome research-4
MI: Myocardial infarction
ECG: Electrocardiography
BBB: Biomarkers, Blood pressure, BIS: Risk Stratification/Management of Patients at Cardiac Risk in Major Noncardiac Surgery
POISE-3: PeriOperative ISchemic Evaluation-3
