# Supplementary material for: Effect of individualized versus conventional perioperative blood pressure management on postoperative major complications in high-risk patients undergoing noncardiac surgery: study protocol for the SPROUT-4 multicenter randomized controlled trial
Source: Trials. 2024 Dec 26;25:850. doi: 10.1186/s13063-024-08707-4 (PMC11673592; doi:10.1186/s13063-024-08707-4)
Supplement: Supplementary file 2 — Additional file 2. English version of model consent form. [file 13063_2024_8707_MOESM2_ESM.docx]

**Appendix. Model Consent Form (English ver.)**

**Note: Each Ethics Committee or Institutional Review Board will revise and adapt according to their own institution’s guidelines.**

| Participant Serial Number |  |
| --- | --- |

**Participant Information Sheet**

**1. Study Title:** Effect of individualized versus conventional perioperative blood pressure management on postoperative major complications in high-risk patients undergoing noncardiac surgery: A multicenter, randomized controlled trial

**2. Principal Investigator:** Karam Nam, Department of Anesthesiology and Pain Medicine

**3. Background and Purpose**

Arterial blood pressure is one of the most basic and critical vital signs and is closely monitored in patients scheduled for surgery under general anesthesia. Intraoperative hypotension can lead to serious postoperative complications such as myocardial injury and acute kidney injury. Therefore, maintaining mean arterial pressure above a certain threshold is a common strategy to prevent intraoperative hypotension. However, there is no clear consensus or standard for defining target blood pressure levels. In general, it is recommended to maintain blood pressure above a universal blood pressure threshold of 55 and 65 mmHg. However, these thresholds have not been established because they are not based on the results of large-scale, multicenter randomized trials; therefore, the thresholds are determined by each clinician in clinical practice.

Therefore, this multicenter, randomized trial was designed to determine whether individualized blood pressure management strategy that maintains mean arterial pressure and systolic blood pressure above -20% of preoperative values would reduce postoperative complications compared with conventional blood pressure management that maintains blood pressure above universal thresholds of mean arterial blood pressure of 65 mmHg and systolic blood pressure of 90 mmHg.

**4. Investigational Medicinal Product/Medical Device**

For each participant, blood pressure during anesthesia will be managed using either an individualized or conventional blood pressure management strategy. Both strategies are commonly used in clinical practice to manage blood pressure during anesthesia. In participants receiving the individualized blood pressure management strategy, mean arterial pressure and systolic blood pressure will be maintained above -20% of their preoperative values, while in participants receiving the conventional blood pressure management strategy, mean arterial pressure and systolic blood pressure will be maintained above 65 and 90 mmHg, respectively, regardless of their preoperative blood pressure level.

This clinical trial is investigator-initiated, and it has not been established whether the use of the individualized blood pressure management strategy, which defines blood pressure thresholds based on preoperative values, is superior to the conventional blood pressure management strategy using universal blood pressure thresholds in the surgical population.

**5. Number of Study Participants and Duration**

A total of 1,980 participants will be enrolled and randomly assigned to the two groups in a 1:1 ratio. The study will last from the start of induction of anesthesia until discharge from the post-operative recovery unit or the end of surgery (if a participant is transferred to the intensive care unit after surgery). During the study period, blood pressure will be recorded for data analysis. All medical care outside the study period will be provided according to the institutional protocol.

**6. Study Procedure and Method**

Surgery and anesthesia will be performed according to institutional protocol, except for a difference in blood pressure management strategies that will be evaluated in this study. Participants will be randomly assigned to the two groups in a 1:1 ratio using a randomization list, and blood pressure will be maintained during the study period using either the individualized or conventional blood pressure management strategy.

During general anesthesia, they will be closely managed and monitored by an attending anesthesiologist. Once a participant enters the operating room and is prepared for surgery, routine monitoring required for surgery and anesthesia will be initiated, general anesthesia will be induced, and participants will be intubated with an endotracheal tube. This is a sequence of general clinical practice that is implemented for all patients undergoing elective surgery, regardless of our clinical trial, without exception.

From the initial administration of general anesthetics until the end of anesthesia, in participants in the individualized blood pressure management group will have their mean arterial pressure and systolic blood pressure maintained above -20% of their preoperative values. In contrast participants in the conventional blood pressure management strategy group will have their mean arterial pressure and systolic blood pressure above 65 and 90 mmHg, respectively. All other procedures, including laboratory testing and medical care will be provided according to institutional protocol, regardless of study participation. There are no additional costs or obligations for participants.

**7. Alternative Treatments (Alternative Treatments besides Those Received as Part of the Study)**

During the study period, medical care for non-cardiac surgery and anesthesia will be provided in the same manner regardless of study participation, except for the blood pressure thresholds. There will be no additional costs. If a participant refuses to participate or is unable to participate in the study, the only alternative option is that the blood pressure control range will be determined by the attending anesthesiologist.

**8. Expected Side Effects, Risks, and Discomforts to Participants**

This trial will investigate postoperative complications by reviewing medical records and laboratory test results. Therefore, the expected risk of adverse events, perioperative risk, discomfort, and unexpected hypersensitivity or adverse reactions to the anesthetic are no different than those experienced by patients undergoing routine non-cardiac surgery and anesthesia.

**9. Anticipated Benefits to Participants**

No direct benefit is expected for participation in the study.

**10. Costs and Compensation**

There is no additional cost or financial compensation to the participant for participation in this study.

**11. Voluntary Participation/Study Discontinuation**

A participant's decision to participate in a clinical trial must be voluntary, and a participant may refuse to participate in a clinical trial or may withdraw from the trial at any time during the trial without loss of any benefit to which the participant may be entitled. If a participant does not meet the inclusion criteria because of a change in the plan, or if the participant develops severe hypotension (if it is deemed difficult to maintain a mean arterial pressure of at least 50-55 mmHg despite the use of institutional protocol), the trial will be discontinued, and appropriate action will be taken. In addition, enrollment may be discontinued in any case where it is deemed difficult for the participant to continue in the study, such as the discovery of a previously unknown serious or underlying medical condition that meets the criteria for exclusion from the study. In addition,

**12. Continuous Provision of New Study-Related Information**

We will promptly notify the participant or the participant's legal representative if we receive new information that may affect our decision about the participant's continued participation in the study.

**13. Injury and Compensation**

Injury related to the clinical trial is not expected, except for minor and temporary pain. However, if unexpected adverse events occur, the injury will be determined to be causally related to participation in the trial and, depending on its severity, the participant will be compensated and provided with appropriate treatment as specified in the protocol for compensation for trial injuries.

**14. Confidentiality**

Any records that could identify participants in this study will be kept confidential, and participant information will be de-identified when the results of the study are published. The results of this study will be used for academic purposes only and may be presented externally for academic reporting. Information from your medical records, such as your gender, age, weight, height, underlying medical conditions, medications, preoperative blood test results, type of surgery, perioperative blood pressure, and postoperative complications, will be used in the study.

However, the participant's participation in this study or the outcome of the participant's treatment will be kept confidential except to the participant, the attending physician, and the study investigators. The participant's medical records will be kept confidential and will not be transferred elsewhere, and may be sent to an review board to review the progress of this trial.

During the trial and even after the trial is completed, the investigator, the investigational committee, institutional review boards, and the Minister of Health and Welfare may have access to the participant's medical records, while maintaining the confidentiality of the participant's identity, in order to review the progress of the trial and the quality of the data in accordance with relevant laws and regulations. Access to the trial records may be authorized by a consent form signed by the participant or the participant's representative. All records will be retained for the legally required period, after which all materials will be securely disposed of.

**15. Contact Information**

Please contact the investigators below for any problems, concerns, or questions arising from the study, and contact Seoul National University Hospital Institutional Review Board or Clinical Research Ethics Center for any problems, concerns, or questions about the rights of human subjects. A copy of the informed consent form will be provided to the subject.

**Principal Investigator**: Karam Nam, Clinical Associate Professor, Department of Anesthesiology and Pain Medicine

**Investigator:** Jae-Woo Ju, Clinical Assistant Professor, Department of Anesthesiology and Pain Medicine

| Participant Serial Number |  |
| --- | --- |

**Participant Informed Consent Form**

**Effect of individualized versus conventional perioperative blood pressure management on postoperative major complications in high-risk patients undergoing noncardiac surgery: A multicenter, randomized controlled trial**

1. I have been given a verbal explanation of the study, read the study description above, and have fully discussed the study with the researcher in charge.

2. I was informed of the risks and benefits of the study, and my questions have been answered to my satisfaction.

3. I voluntarily agree to participate in this study.

4. I understand that I can refuse to participate in the study or withdraw from the study at any time without affecting my future medical care and such a decision will not harm me in any way.

5. By signing this information sheet and informed consent form, I agree that my personal information will be collected and handled by the researcher for medical research purposes to the extent that current law and regulations permits.

* Personal information collected for this study will be stored for 3 years from the time the study ends according to relevant legislation.

6. I understand that I will receive a copy of this study information sheet and the informed consent form.

|  |  |  |  |  |
| --- | --- | --- | --- | --- |
| Name of Participant |  | Signature |  | Date |
|  |  |  |  |  |
| Name of Investigator/Researcher |  | Signature |  | Date |
|  |  |  |  |  |
| Name of Legally Authorized Representative |  | Signature |  | Date |
|  |  |  |  |  |
| (Relationship with representative) |  | (Reason for obtaining representative’s consent) |  |  |
